# In Vitro Evaluation of *Weizmannia coagulans* Strain LMG S-31876 Isolated from Fermented Rice for Potential Probiotic Properties, Safety Assessment and Technological Properties

**DOI:** 10.3390/life12091388

**Published:** 2022-09-06

**Authors:** Madapati Sreenadh, Kallur Ranjith Kumar, Soumitra Nath

**Affiliations:** 1 Abode Biotec India Private Limited, MLA Colony, Banjara Hills, Hyderabad 500033, Telangana, India; 2 Department of Biotechnology, Gurucharan College, Silchar 788004, Assam, India

**Keywords:** probiotic, fermented rice, *Weizmannia* *coagulans*, artificial gastric juice, antibiotic, lipolytic activity

## Abstract

**Simple Summary:**

*Weizmanniacoagulans* strain LMG S-31876, isolated from fermented rice, is Gram-positive bacilli, a spore-forming, motile, and facultative anaerobe, with an optimum temperature requirement of 40 °C. The strain is able to tolerate acidic gastric juice, bile, and pancreatin. It is non-virulent and exhibits sensitivity to most of the tested antibiotics. The strain shows antagonistic activity against pathogenic bacteria. The 16S rDNA gene sequence of *W.* *coagulans* strain LMG S-31876 has been submitted to NCBI–GenBank, archiving accession number MZ687045. The strain has also been deposited to BCCM/LMG and MTCC-IDA with reference numbers LMG S-31876 and MTCC 25396, respectively.

**Abstract:**

*Bacillus coagulans*, which has been taxonomically reclassified as *Weizmannia coagulans*, has been the focus of research due to its wide distribution in fermented foods, probiotic properties, and tolerance to extreme environments. The purpose of this study was to characterise putative probiotic bacteria in a fermented rice sample, followed by an in vitro screening of presumptive probiotic properties and a safety assessment to ensure their safety for human consumption. The predominant isolate was Gram-positive, rod-shaped, catalase-positive, spore-forming, motile, and facultatively anaerobic. The biochemical test and 16S rDNA sequencing identify the isolate as *Weizmannia coagulans* strain LMG S-31876. The strain showed significant viability in acidic gastric juice, pancreatin, and bile. The strain showed tolerance to 5% NaCl, and a low-to-moderate percentage of hydrophobicity and auto-aggregation was recorded. It met all safety criteria, including haemolytic activity, DNase activity, antibiotic sensitivity, and growth inhibition of other bacteria. Evaluation of its technological properties showed positive results for amylolytic and lipolytic activities; however, negative results were obtained for proteolytic activity. It could be concluded from the gathered data that *W. coagulans* strain LMG S-31876 isolated from fermented rice, might serve as a potential functional probiotic food. However, extended follow-up durations and larger-scale trials by assessing the therapeutic effects in managing various clinical gastrointestinal conditions are required to warranty such effects.

## 1. Introduction

Over the past few years, *Bacillus coagulans* has become a research centre due to its probiotic characteristics and high tolerance to extreme environments. The Gram-positive bacterium *B. coagulans* is aerobic or facultatively anaerobic and spore-forming. In the fifth edition of Bergey’s Manual, *B. coagulans* was described as *Lactobacillus sporogenes* due to its ability to produce lactic acid [1]. A detailed investigation of its physiological and biochemical properties led to its reclassification in Bergey’s Manual’s seventh edition under the genus *Bacillus* [2]. However, recent phylogenomic analysis suggests the reclassification of *Bacillus coagulans* within the genus *Weizmannia* and it has been taxonomically reassigned as *Weizmannia coagulans* [3].

*Weizmanniacoagulans* is naturally distributed in various fermented foods prepared from rice, maize, cabbage, soybean, fruit juice, milk, meat, etc. [4,5,6,7]. There is evidence that probiotic strains of *W. coagulans* can safely be used to treat major depression with irritable bowel syndrome (IBS) and relieve the clinical symptoms of IBS, such as vomiting, diarrhoea, and irregular bowel movements [8,9]. The benefits also include improving bloating and abdominal pain [10], as well as maintaining a healthy microbiome in the human digestive system [11], recovering exercise-induced muscle damage [12], relieving respiratory tract symptoms [13], preventing bacterial vaginosis [14], and inhibiting the growth of cancer cells [15]. Consequently, *W. coagulans* is being commercialised as various health supplements, such as LactoSpore^®^, SIBNO™, GanedenBC30, PROBC, etc. [12,13,16,17]. These strains are Generally Recognized as Safe (GRAS) and have Qualified Presumptions of Safety (QPS) according to FDA and EFSA guidelines.

Probiotic strains commonly found in foods and health supplements are expected to survive in the gastrointestinal tract by overcoming the stress induced by acidic gastric acid, bile, and pancreatin [18]. The extracellular enzymes produced by *W. coagulans* are capable of degrading large biomolecules into smaller subunits [19,20]. A probiotic strain must also adhere to intestinal cells and exhibit antagonistic activity against pathogenic bacteria [21]. The genomic investigation of *W. coagulans* revealed that antibiotic resistance genes were not readily transferable to other bacteria and that no additional genes posed significant safety risks [22]. In this study, a novel strain of *W. coagulans* was isolated from fermented rice, followed by an in vitro evaluation of the presumptive probiotic properties, safety, and technological parameters.

## 2. Materials and Methods

### 2.1. Isolation, Identification and Safe Deposit of Bacteria

#### 2.1.1. Isolation of Bacteria

White rice samples approximately of 80 g were cooked with water for around 30 min to maintain a proper consistency. Subsequently, fermentation was carried out by cooking rice in distilled water with a ratio of 1:3 for 12–18 h, maintaining at a temperature of 27 °C [23]. Fermented rice samples were serially diluted (10^−4^–10^−6^ fold) with distilled water, and thereafter, 0.1 mL of aliquot was poured on freshly prepared deMan Rogosa Sharpe (MRS) medium (Himedia, Mumbai, India). The MRS plates were then incubated at 35–40 °C for a period of 24–48 h [24,25]. In order to obtain pure cultures, the individual colonies were sub-cultured on freshly prepared MRS agar plates.

#### 2.1.2. Morphological and Biochemical Characterisation

The preliminary identification of the isolate was based on the culture characteristics, colony morphology, and microscopic observations. Biochemical tests were used to screen Gram-positive bacilli that form spores, including the indole test, methyl red test, Voges-Proskauer test, citrate utilisation test, catalase test, oxidase test, and starch hydrolysis test [26].

#### 2.1.3. Molecular Identification

Genomic DNA was isolated by following the protocol of Green, Hughes [27]. The 16S rDNA region of isolated DNA was amplified using 27F forward primer (5′-AGA GTT TGA TCM TGG CTC AG-3′) and 1492R reverse primer (5′-TAC GGY TAC CTT GTT ACG ACT T-3′) obtained from Sigma-Aldrich, Bangalore, India. The amplified gene was sequenced at Macrogen (Seoul, Korea) using the ABI 3730xl 96 capillary system equipped with the Big Dye Terminator v3.1 kit. For sequencing, 785F (5′-GGA TTA GAT ACC CTG GTA-3′) and 907R (5′-CCG TCA ATT CMT TTR AGT TT-3′) primers were used. After sequencing the genes, a consensus sequence of 16S rDNA was generated using the Geneious R8 software package (Biomatters Ltd., Auckland, New Zealand), followed by BLAST analysis to find homologous sequences in non-redundant databases. A total of ten best-match sequences were selected from the database following the highest similarity and maximum identity scores. A neighbor-joining tree was constructed based on the alignment of the sequences using Clustal-W [23,28]. 

#### 2.1.4. Safe Deposit

The isolated strain has been deposited in Belgian Coordinated Collections of Microorganisms/Laboratorium voor Microbiologie (BCCM/LMG) and Microbial Type Culture Collection–International Depositary Authority (MTCC–IDA) under the Budapest Treaty.

### 2.2. Evaluation of Probiotic Properties

#### 2.2.1. Test for Resistance to Low pH

In the stomach, the food remains in an acidic environment for at least three hours, so initial cell viability is influenced by pH. To determine the pH tolerance of the test isolate, 500 µL of bacterial culture in MRS broth was inoculated in separate test tubes containing 5 mL of PBS solution. The test tubes were pre-adjusted at pH 3.0 and 7.20 with 1 N HCl and 1 N NaOH. The tubes were incubated at 40 °C for 4 h. The total number of viable colonies was measured at every 1 h interval by spreading 100 µL bacterial suspension on MRS agar plates. The result was expressed in log_10_ colony-forming units per millilitre (log_10_ CFU/mL).

#### 2.2.2. Simulated Gastric Juice Tolerance Test

In total, 100 µL of overnight-grown bacterial culture in MRS broth was centrifuged for 15 min at 2000× *g* force to collect the cell pellet. The pellets were suspended in 10 mL of simulated gastric juice (assay) and incubated at 40 °C for 4 h. Simulated gastric juice was prepared by using 3 g/L pepsin, 7 mM KCl, 45 mM NaHCO_3_, and 125 mM NaCl, with the pH adjusted to 3.0 [29]. The cell pellets were also inoculated in PBS at neutral pH and kept for incubation as a control. The viable cell count in both the assay and control was determined at regular intervals by plating 100 µL of samples on fresh MRS agar plates. 

#### 2.2.3. Bile Tolerance Test

The bile tolerance test was carried out following the method described by Nath, Sikidar [30]. In total, 100 µL of bacterial culture in MRS broth was inoculated in MRS broth containing 0.3% ox-bile salts and incubated at 40 °C for 4 h. The bacterial culture was also inoculated into bile-free MRS broth, which serves as a control. The viable cell count of the bacterial isolate was determined hourly by spreading 100 µL of samples onto fresh MRS agar plates. 

#### 2.2.4. Pancreatin Tolerance Test

In order to determine the pancreatin tolerance, 100 µL of bacterial culture in MRS broth was inoculated into 10 mL of MRS broth containing 0.5% (*w*/*v*) pancreatin. A control set was prepared by inoculating the bacterial suspension in pancreatin-free MRS broth. Both the assay and control tubes were kept in an incubator at 40 °C for 48 h. The number of viable bacterial cells at 24 h and 48 h was determined and expressed as log_10_ CFU/mL [31]. 

#### 2.2.5. Lysozyme Tolerance

A lysozyme tolerance test was performed in MRS broth supplemented with different lysozyme concentrations. The lysozyme concentration in the broth was maintained at 100, 200, and 300 mg/L. The broth without lysozyme was used as a control. Thereafter, 1 mL of overnight-grown bacterial culture in MRS broth was inoculated in 10 mL of the broth and incubated at 40 °C for 3 h. The viable cells were enumerated on MRS agar plates, and the results were expressed as log_10_ CFU/mL [32].

#### 2.2.6. Cell Surface Hydrophobicity

The capability of the bacteria to stick with hydrocarbons, known as cell surface hydrophobicity, could be indicative of the extent of adhesion to the epithelial cells in the gastrointestinal tract [33]. To test this, overnight-grown bacterial culture in MRS broth was centrifuged at 2000× *g* force for 10 min at 4 °C to recover the bacterial pellets. Using 6 mL of PBS solution (pH 7.2), the cell pellets were washed twice and resuspended in PBS to measure the initial absorbance at 600 nm. Thereafter, 3 mL of the bacterial suspension was added to 1 mL of hydrocarbons (n-Hexadecane and Toluene) and mixed by vortexing for 2 min. For phase separation, the suspension was left undisturbed for one hour, followed by the careful removal of the aqueous phase. The remaining volume was used to record the final absorbance (OD_final_) [34]. The initial and final absorbance was used to measure the percentage of cell surface hydrophobicity by the formula:

$$\mathrm{Percentage}\;\mathrm{Hydrophobicity}\;\left(\%\right)=\frac{{\mathrm{OD}}_{\mathrm{initial}}-{\mathrm{OD}}_{\mathrm{final}}}{{\mathrm{OD}}_{\mathrm{initial}}}\times 100$$

#### 2.2.7. Cellular Autoaggregation

The specific cell–cell interaction or autoaggregation was determined by the method described by Xu, Jeong [35]. The cell pellets from an overnight-grown culture in MRS broth were harvested by centrifugation at 2000× *g* force for 10 min at 4 °C. The pellets were washed twice with PBS solution and resuspended in 6 mL of the same solution. A 600 nm absorbance measurement was taken to determine the initial absorbance (OD_initial_). Afterwards, the cells were incubated at 40 °C for 2 h, and the final absorbance (OD_final_) was measured to determine the cellular autoaggregation percentage.

$$\mathrm{Percentage}\;\mathrm{Aggregation}\;\left(\%\right)=\frac{{\mathrm{OD}}_{\mathrm{initial}}-{\mathrm{OD}}_{\mathrm{final}}}{{\mathrm{OD}}_{\mathrm{initial}}}\times 100$$

#### 2.2.8. NaCl Tolerance Test

The test isolate was streaked on MRS agar plates supplemented with varied NaCl concentrations [0.5, 1, 2, 3, … 10% (*w*/*v*)] [23]. A control plate was created with MRS agar without NaCl amendment. All of the plates were incubated at 40 °C, and their growth pattern was observed after 24–72 h.

### 2.3. Safety Assessment

#### 2.3.1. Haemolytic Activity

A haemolytic test was performed on blood agar plates, which were prepared by supplementing 5% sheep blood (obtained from a local butcher shop) on Brain Heart Infusion (BHI) agar. The test isolate was streaked onto the surface of freshly prepared blood agar plates. The plates were incubated at 40 °C for 24–48 h, and the haemolysis pattern (α, β, and γ) was noted.

#### 2.3.2. DNase Activity

The test isolate was streaked on DNase agar plates (Himedia, Mumbai, India) and incubated anaerobically at 40 °C for 72 h [36]. The plates were then flooded with 3% HCl for 8 min, and the colonies were examined for a clear zone.

#### 2.3.3. Antibiotic Resistance

The antibiotic susceptibility test was conducted using the Kirby–Bauer disk diffusion method [37]. The standard antibiotic discs were procured from ‘HiMedia, Mumbai, India, which include polymyxin-B (300 µg), amoxiclav (30 µg), rifampicin (5 µg), tetracycline (30 µg), oxacillin (5 µg), amikacin (30 µg), cefoxitin (30 µg), cefepime (30 µg), ceftazidime (30 µg), cefotaxime (30 µg), chloramphenicol (30 µg), cefdinir (5 µg), penicillin g (10 µg), moxifloxacin (5 µg), ampicillin (10 µg), vancomycin (30 µg), ceftriaxone (30 µg), neomycin (10 µg), ofloxacin (5 µg), norfloxacin (10 µg), kanamycin (30 µg), bacitracin (10 µg), co-trimoxazole (25 µg), methicillin (10 µg), streptomycin (10 µg), levofloxacin (5 µg), erythromycin (15 µg), clindamycin (2 µg), gentamycin (120 µg), and sterile disc (control). The antibiotic discs were placed onto the freshly prepared lawns of each isolate on Mueller–Hinton agar (MHA) plates. The plates were incubated at 40 °C for 24–48 h, and the diameter of the zone of inhibition was measured in millimetres. The strains were classified in accordance with the Clinical and Laboratory Standards Institute [38], following the standard antibiotic disc chart.

#### 2.3.4. Extraction of Antibacterial Agents and Evaluation of Their Antagonistic Activity

The isolation of the antibacterial agents was performed according to the protocol of Hussein, Jacob [39]. The overnight-grown bacterial broth was added to an equal volume of ethyl acetate and kept in a rotary shaker at 20 rpm for 10 min. After mixing, the suspension was centrifuged, and the supernatant was collected in a fresh tube. The supernatant containing ethyl acetate was allowed to evaporate, and the remaining contents were used to study the antagonistic effect.

The well-diffusion method was used to investigate the efficacy of bacterial metabolites [40]. To perform this, wells were created with a cork borer in freshly prepared MHA plates. Thereafter, the suspension of each test pathogen was spread onto the Petri plates using a sterile spreader, and 60 µL of bacterial extracts was filled into the wells. The control well was filled with Dimethyl sulfoxide (DMSO). After incubation at 40 °C for 24–48 h, the zone of inhibition was measured.

The test pathogens were obtained from the culture collection of Institutional Biotech Hub, Gurucharan College, Silchar, which includes *Staphylococcus aureus* strain GCC_20MS, *Mammaliicoccus sciuri* strain GCC_20RS, *Bacillus cereus* strain GCC_21R1, *Bacillus nealsonii* strain GCC_21R8, *Bacillus megaterium* strain GCC-SO1, *Enterobacter bugandensis* strain GCC_21R10, *Pseudomonas aeruginosa* strain GCC_19W1, *Stenotrophomonas maltophilia* strain GCC_19W2, *Achromobacter spanius* strain GCCSB1, and *Acinetobacter johnsonii* strain SB_SK.

### 2.4. Evaluation of Technological Properties

#### 2.4.1. Proteolytic Activity

Proteolytic activity was carried out on an agar medium composed of skimmed milk powder (10% *w*/*v*) and agar (2% *w*/*v*), in which wells were formed with a cork borer [41]. Twenty microlitres of the bacterial suspension were added to the wells, and the plate was incubated at 40 °C for 72 h. The formation of a clear zone around the colonies indicates a positive result for proteolytic activity.

#### 2.4.2. Lipolytic Activity

Test isolate was evaluated for lipolytic activity according to the method described by Aspri, Bozoudi [42]. The isolate was streaked on tributyrin agar plates and incubated at 40 °C for 96 h. Positive results for lipolytic activity are indicated by clear halos surrounding the colonies.

#### 2.4.3. Amylolytic Activity

The amylolytic activity was determined by spotting the test isolate onto the surface of Luria Burtani (LB) agar containing 20 g/L of soluble starch, followed by incubation at 37 °C for 72 h. The LB plate was flooded with iodine solution (1% *w*/*v*). The appearance of a halo zone around the colonies confirms positive results for amylolytic activity [43]. 

### 2.5. Statistical Analysis

All experiments were performed in triplicates, and the statistical analysis was carried out using Microsoft Excel 2007 and SPSS version 16. The viable plate counts are expressed as the mean ± standard deviation of log_10_ colony-forming units per millilitre (log_10_ CFU/mL). Statistical comparisons were drawn using the t-test, and differences were considered statistically significant at *p* < 0.01 and *p* < 0.05.

## 3. Results

### 3.1. Identification and Safe Deposit of Bacteria

#### 3.1.1. Morphological and Biochemical Characteristics

The isolated bacterial strain characterised in the present study resulted in being Gram-positive bacilli that are spore-forming and facultatively anaerobic. On MRS agar plates, the colonies were elevated, small-medium sized, and white/creamy in colour without pigmentation. The cell size has been found to be 0.9 by 3.0 to 5.0 μm, present singly or in-chain. The strain showed positive results in the indole test, methyl red test, Voges–Proskauer test, catalase test, oxidase test, and starch hydrolysis test (Table 1). Based on the gathered results, the isolated strain LMG S-31876 was presumed as *Weizmannia coagulans.*

#### 3.1.2. Molecular Characterisation and Phylogenetic Analysis 

**Figure 1:**
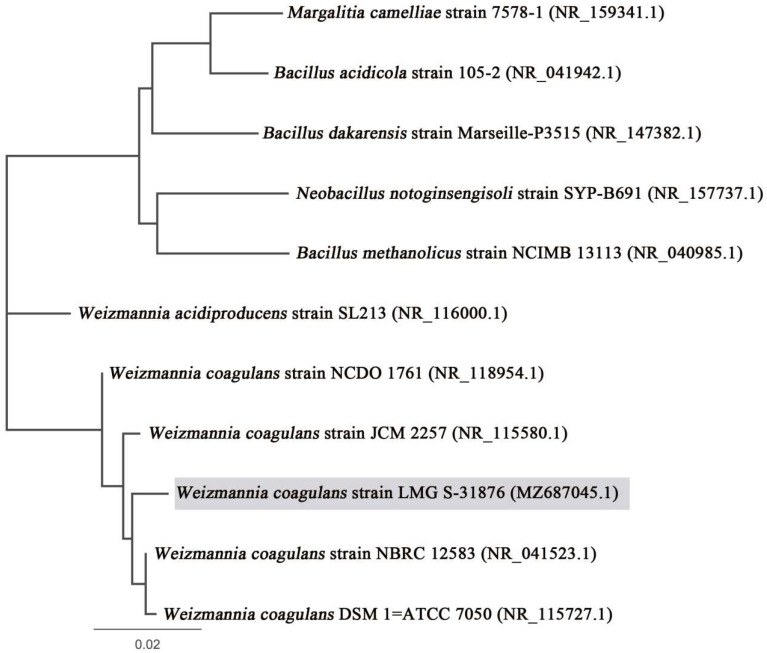
Phylogenetic tree of *Weizmannia* *coagulans* strain LMG S-31876, based on 16S rDNA gene sequence. The tree was constructed by the neighbour joining method, using Geneious R8 software package.

**Figure 2:**
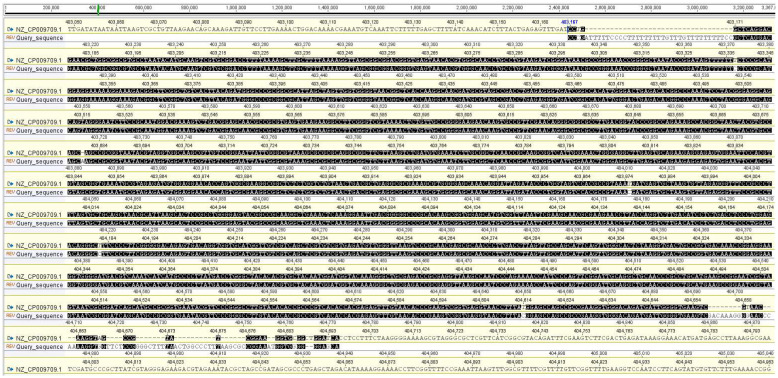
Alignment of 16S rDNA gene sequence of *W. coagulans* LMG S-31876 (Accession Number: MZ687045) with the complete genome of *W. coagulans* DSM 1 = ATCC 7050 (Accession Number: NZ_CP009709.1).

The closest homologue of the isolate LMG S-31876 was found to be *Weizmannia* *coagulans* DSM 1 = ATCC 7050 (accession number: NR_115727.1) (Figure 1). Based on phylogenomic analysis and recent bacterial classification, the isolate was taxonomically assigned as *Weizmannia coagulans* strain LMG S-31876. The 16S rDNA gene sequence of the isolated strain has been submitted to NCBI–GenBank, archiving accession number MZ687045. Bowtie 2 was used to align the 16S rDNA gene sequence of *W. coagulans* strain LMG S-31876 with the complete genome of *W. coagulans* DSM 1 = ATCC 7050 (accession number: NZ_CP009709.1) and constructed a reference map using Geneious R8 (Biomatters Ltd., Auckland, New Zealand), which is represented in Figure 2.

#### 3.1.3. Safe Deposit and Accession Number

The isolated strain of *W. coagulans* was submitted for safe deposit with the reference code ProBC Plus. The accession numbers obtained from BCCM/LMG and MTCC-IDA are LMG S-31876 and MTCC 25396, respectively. 

### 3.2. Evaluation of Probable Probiotic Properties

#### 3.2.1. Test for Resistance to Low pH

**Figure 3:**
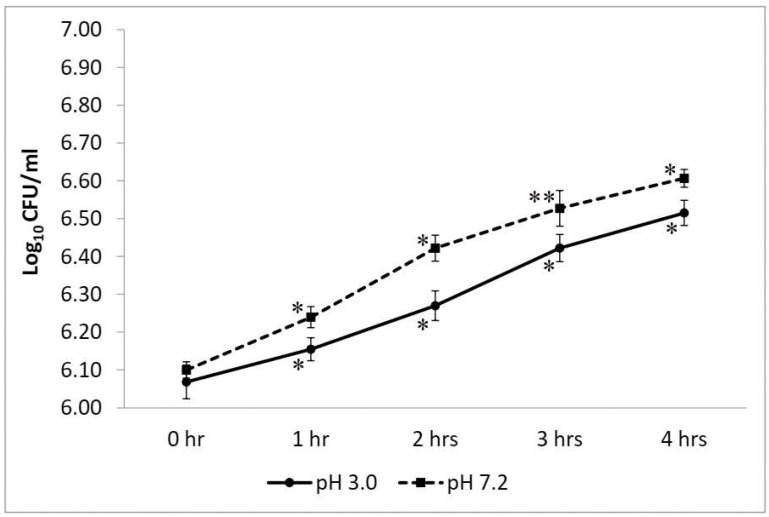
Viable plate count of *W. coagulans* strain LMG S-31876 at pH 3.0 and pH 7.2. The results are expressed as mean ± standard deviation of log_10_ CFU/mL of three replicates. “**” and “*” stand for the significance level of *p* < 0.01 and *p* < 0.05, respectively.

*W. coagulans* strain LMG S-31876 showed remarkable growth at pH 3.0 without any significant loss in viability. Compared to the control set at pH 7.2, the survival rate of *W. coagulans* strain LMG S-31876 at pH 3.0 was statistically significant at *p* < 0.05. At acidic pH (pH 3.0), the isolate exhibited an initial lag phase with viability of 6.15 ± 0.03 log_10_ CFU/mL at 1 h, which increased to 6.52 ± 0.03 log_10_ CFU/mL at 4 h. The maximum mean difference (*p* < 0.01) and an exponential increase in the bacterial count were recorded from 2 and 3 h at pH 3.0; whereas at pH 7.2, a significant increase (*p* < 0.05) was evidenced between 1 to 2 h (Figure 3).

#### 3.2.2. Simulated Gastric Juice Tolerance Test

**Figure 4:**
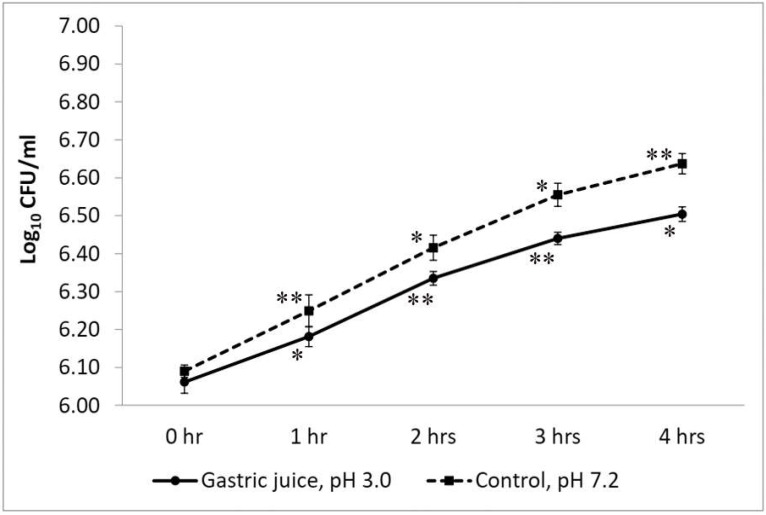
Viable plate count of *W. coagulans* strain LMG S-31876 in the presence of simulated gastric juice (pH 3.0) and control (pH 7.2). The results are expressed as mean ± standard deviation of log_10_ CFU/mL of three replicates. “**” and “*” stand for the significance level of *p* < 0.01 and *p* < 0.05, respectively.

The isolated strain of *W. coagulans* showed significant growth in simulated gastric juice (at pH 3.0) as compared with the control set at pH 7.0 (Figure 4). The initial count of LMG S-31876 in acidic gastric juice was 6.06 ± 0.03 log_10_ CFU/mL, which, after 1 h of incubation, increased significantly (*p* < 0.01) to 6.44 ± 0.20 log_10_ CFU/mL. Upon further incubation up to 4 h, the viable bacterial count marginally increased to 6.44 ± 0.02 log_10_ CFU/mL. The viability of LMG S-31876 in the control set was significant at *p* < 0.05.

#### 3.2.3. Bile Tolerance Test

**Figure 5:**
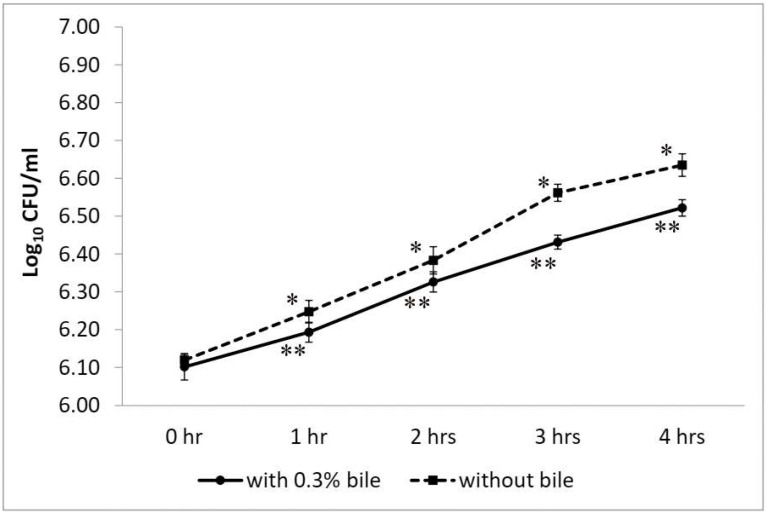
Viable plate count of *W. coagulans* strain LMG S-31876 in the presence of 0.3% bile compared with the control set without bile. The results are expressed as mean ± standard deviation of log_10_ CFU/mL of three replicates. “**” and “*” stand for the significance level of *p* < 0.01 and *p* < 0.05, respectively.

*W. coagulans* strain LMG S-31876 showed tolerance to 0.3% bile and exhibited significant (*p* < 0.01) cell viability during the 4 h incubation period. The initial bacterial count in 0.3% bile was 6.10 ± 0.03 log_10_ CFU/mL, which exponentially increased to 6.52 ± 0.02 log_10_ CFU/mL at 4 h (Figure 5). The control set (without bile) also showed remarkable growth, recording an initial viable count of 6.12 ± 0.02 and reaching 6.25 ± 0.03, 6.38 ± 0.04, and 6.56 ± 0.02 log_10_ CFU/mL after 1, 2, and 3 h, respectively. Further incubation up to 4 h recorded a viable bacterial count of 6.64 ± 0.03 log_10_ CFU/mL that showed a marginal decrease in the mean difference. Statistically, the viable plate count of *W. coagulans* strain LMG S-31876 was found to be significant (*p* < 0.05) for all datasets.

#### 3.2.4. Pancreatin Tolerance Test

**Figure 6:**
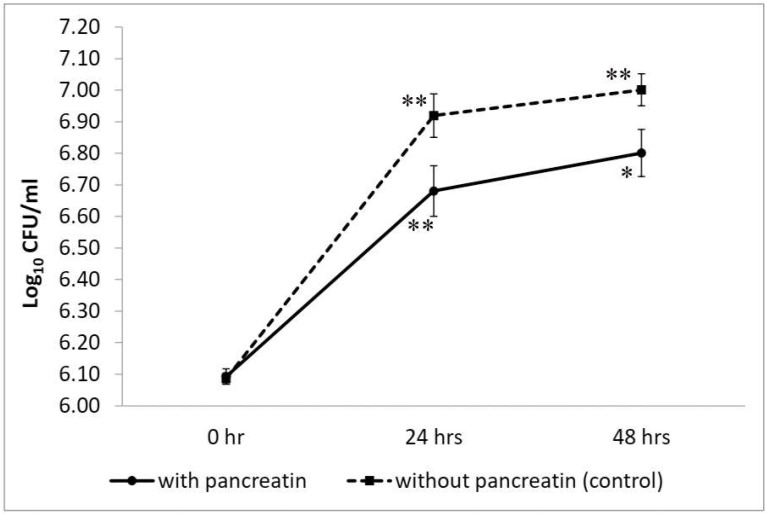
Viable plate count of *W. coagulans* strain LMG S-31876 in the presence of 0.5% pancreatin, and comparison with the control set without pancreatin. The results are expressed as mean ± standard deviation of log_10_ CFU/mL of three replicates. “**” and “*” stand for the significance level of *p* < 0.01 and *p* < 0.05, respectively.

*W. coagulans* strain LMG S-31876 showed considerable growth and viability in pancreatin-containing MRS broth at 24 h of incubation. The initial count of the test isolate in MRS broth was 6.09 ± 0.02 log_10_ CFU/mL. In the presence of pancreatin, *W. coagulans* strain LMG S-31876 showed 6.68 ± 0.08 log_10_ CFU/mL viable count at 24 h. However, upon further incubation up to 48 h in pancreatin-containing MRS broth, the isolate failed to exhibit further growth and showed a viable count of 6.80 ± 0.07 log_10_ CFU/mL. The control set without pancreatin showed a sharp increase in bacterial viability during the entire incubation period, recording 6.92 ± 0.07 and 7.00 ± 0.05 log_10_ CFU/mL at 24 h and 48 h, respectively (Figure 6).

#### 3.2.5. Lysozyme Tolerance Test

The initial bacterial count of *W. coagulans* strain LMG S-31876 was 6.40 ± 0.01 log_10_ CFU/mL, and the survival rate proportionally increased with the decreasing Lysozyme concentration. At 100 mg/L Lysozyme concentration, the viable count reached 6.67 ± 0.02 log_10_ CFU/mL at 3 h, which was found to be significant at *p* < 0.01. With the further increase in Lysozyme concentrations to 200 mg/L, a significant (*p* < 0.05) viability was recorded at 3 h. However, upon a further increase to 300 mg/L, a non-significant (*p* > 0.05) viability was observed.

#### 3.2.6. Cell Surface Hydrophobicity

*W. coagulans* strain LMG S-31876 showed low-to-moderate hydrophobicity to the hydrocarbons, showing 13.03% for n-hexadecane and 17.04% for toluene. 

#### 3.2.7. Cellular Autoaggregation

The cellular autoaggregation of *W. coagulans* strain LMG S-31876 was found to be 18.27%, indicating its moderate ability to colonise and attach to the intestinal epithelium.

#### 3.2.8. NaCl Tolerance Test

*W. coagulans* strain LMG S-31876 showed significant growth in 5% NaCl after 24 h of incubation. The cell viability in the culture plate was significantly reduced with the amendment of 6% NaCl. However, upon a further increase in NaCl concentrations (7% and above), no visible growth was observed, even after 72 h of incubation.

### 3.3. Safety Assessment

#### 3.3.1. Haemolytic Activity

*W. coagulans* strain LMG S-31876 showed no reaction in the surrounding areas of blood, which is considered γ-haemolysis.

#### 3.3.2. DNase Activity

There was no clear zone around the colonies of the test isolate after flooding the DNase agar plates with 3% HCl, indicating no DNase activity.

#### 3.3.3. Antibiotic Sensitivity and Resistance Pattern

*W. coagulans* strain LMG S-31876 was susceptible to most of the tested antibiotics (Table 2). The highest sensitivity was observed against tetracycline (44 mm), amoxiclav (42 mm), methicillin (37 mm), cefdinir (37 mm), and gentamycin (36 mm). Moderately low sensitivity was observed against penicillin-G, co-trimoxazole, and erythromycin, which had zones of inhibition of 18, 13, and 11 mm, respectively.

#### 3.3.4. Extraction of Antibacterial Agents and Evaluation of Their Antagonistic Activity

The antimicrobial agents of *W. coagulans* strain LMG S-31876 inhibited the growth of all test pathogens (Table 3). A zone of inhibition greater than 20 mm was observed against *Staphylococcus aureus*, *Pseudomonas aeruginosa*, and *Achromobacter spanius* which are infectious to humans. The metabolites of *W. coagulans* also potently inhibit the growth of *Mammaliicoccus sciuri* and *Acinetobacter johnsonii*, which were isolated from infected skin samples. The tested *Weizmannia* sp. showed moderate inhibition against other *Bacillus* species (10–14 mm), which are reportedly found in the polluted environment and rarely colonise the human gastrointestinal tract.

### 3.4. Evaluation of Technological Properties

#### 3.4.1. Proteolytic Activity

*W. coagulans* strain LMG S-31876 showed negative results for proteolytic activity, indicated by the absence of any clear zone around the colonies after incubation in skim milk agar medium.

#### 3.4.2. Lipolytic Activity

The strain showed a positive result for the lipolytic test, evidenced by a clear halo zone around the colonies.

#### 3.4.3. Amylolytic Activity 

*W. coagulans* strain LMG S-31876 showed a halo zone around the colonies upon flooding the agar plates with iodine solution (1% *w*/*v*), indicating positive results for the amylolytic test.

## 4. Discussion

The isolated strain LMG S-31876 was a Gram-positive rod, spore-forming, with an optimal growth temperature of 40 °C. Biochemical tests showed positive results for the indole test, methyl red test, Voges–Proskauer test, catalase test, oxidase test, and starch hydrolysis test, which was consistent with other studies [11,44]. Sequence analysis confirmed clustering with *Weizmannia coagulans* DSM 1 = ATCC 7050, which shares 99% sequence homology. The 16S rDNA sequence was submitted to NCBI-GenBank, archiving accession number MZ687045. The safe deposit accession number of *W. coagulans* in BCCM/LMG is LMG S-31876, and for MTCC-IDA it is MTCC 25396. 

The beneficial effects of probiotics are dependent on their resistance to the gastrointestinal environment, which was taken into account during in vitro testing. The present study showed that *W. coagulans* strain LMG S-31876 could significantly tolerate low pH, gastric juice, bile and pancreatin, thereby efficiently withstanding the passage of the gastrointestinal tract. The strain survived at pH 3.0, achieving significant (*p* < 0.05) viability up to 3 h. Similar results were also noted by the probiotic strains of *W. coagulans* IDCC 1201 [7], *W. coagulans* GBI-30, 6086 [45], and *W. coagulans* BDU3 [44]. The viability of *W. coagulans* strain LMG S-31876 in acidic gastric juice is similar to that of other probiotic strains commonly found in traditional foods and dairy products [23,30,46,47,48,49]. Bile salts in the gastrointestinal tract exert an antimicrobial effect, which in most cases reduces bacterial viability. Thus, probiotic microorganisms should remain viable in acidic and bile-salt environments [50]. As compared with previously reported *W. coagulans* strains [7,45], our study demonstrated a better survival rate by *W. coagulans* strain LMG S-31876, exhibiting significant (*p* < 0.05) viability at 0.3% bile and pancreatin. Bernet-Camard, Coconnier [51] reported that the human intestinal epithelial cells secrete lysozyme, which exerts an antimicrobial effect and acts as a protective physical barrier. Lysozyme resistance is, therefore, an important criterion in probiotic strain selection. It has been observed that *W. coagulans* strain LMG S-31876 was highly resistant to lysozyme, which is significant to the findings of other studies [45]. The probiotic strain of *Bacillus* spp. isolated from fermented pickles and idli has been found to tolerate 100 μg/mL lysozyme concentrations [32,52].

In order to colonise the intestinal tract, probiotic strains must have hydrophobic cell surfaces and be capable of autoaggregation [53]. *W. coagulans* strain LMG S-31876 displayed 13.03% and 17.04% hydrophobicity for the hydrocarbons n-hexadecane and toluene, respectively, and the cellular autoaggregation was found to be 18.27%. Probiotic bacteria, including *Lactobacillus* spp. and *Bacillus* spp., have been shown to have hydrophobicities ranging from 6.1 to 87.4% [53,54,55]. In agreement with our findings, Bang, Ban [7] reported a hydrophobicity of 17.5% and autoaggregation ability of 13.0–29.1% for *W. coagulans* IDCC 1201. Although *W. coagulans* strain LMG S-31876 showed remarkable growth in 5% NaCl, its viability decreases with further increase in NaCl concentration.

*W. coagulans* strain LMG S-31876 exhibited γ-haemolysis and an inability to produce DNase, ensuring its non-virulent nature, which may be regarded as a selection criterion for potential probiotic strains [7,56]. Results showed that the isolate was susceptible to most antibiotics, thus preventing the spread of antibiotic resistance to the gut microbiota [57]. Similar to our findings, many researchers have also reported the sensitivity pattern of *W. coagulans* towards antibiotics [45]. Numerous studies have also reported that *W. coagulans* lacks mobile elements associated with antibiotic resistance genes [7,22,58]. Another crucial property of all potential probiotic strains is their antimicrobial activity against pathogens. *W. coagulans* strain LMG S-31876 showed growth inhibition in all tested bacteria, exhibiting the maximum zone against *Staphylococcus aureus*, *Pseudomonas aeruginosa, Achromobacter spanius*, *Mammaliicoccus sciuri*, and *Acinetobacter johnsonii*. In agreement with the findings of other studies, probiotic strains of *W. coagulans* exhibit antagonistic activity against both Gram-positive and Gram-negative bacteria [45,59,60]. 

The technological parameters of *W. coagulans* strain LMG S-31876 showed its ability to produce extracellular amylolytic and lipolytic enzymes. These microbial enzymes can be industrially used for various applications, including medicines, detergents, cosmetics, food flavours, industrial fermentation, and waste treatment [61,62,63,64]. Microbial enzymes derived from bacteria are more valuable than those derived from animals or plants because of the vast diversity of catalytic activities, more stability, ease of genetic manipulation, rapid growth, high yields, and less-expensive media requirements [62].

## 5. Conclusions

*Weizmanniacoagulans* strain LMG S-31876 is a potential probiotic bacterial strain, considering its tolerance to acidic gastric juice, bile, pancreatin, and lysozyme, as well as its cell surface hydrophobicity and autoaggregation abilities. The strain also fulfilled safety criteria concerning its non-virulence nature, antibiotic sensitivity, and antagonistic activity against pathogenic bacteria. It also showed technological parameters by producing extracellular amylolytic and lipolytic enzymes that can be used in a wide variety of clinical applications. Thus, the excellent viability during digestion, high adhesive capacity, and non-virulent nature of *W. coagulans* strain LMG S-31876 means it is a suitable candidate for starter cultures and pharmaceutical formulations. However, extended follow-up durations and larger-scale trials by assessing the therapeutic effects in managing various clinical gastrointestinal conditions are required to warranty such effects.

## Figures and Tables

**Table 1:** Morphological and biochemical characteristics of the isolated bacteria.

Parameters	Observations
**General Characteristics**	
MRS broth	Turbid
MRS agar	Elevated, small-medium-sized colonies
Colony colour	White and creamy
Pigmentation	No
Growth temperature	40 °C
Growth condition	Facultative anaerobe
Cell arrangement	Single or in-chain
Cell shape	Bacillus
Gram staining	Gram-positive
Endospore test	Positive
**Biochemical Characteristics**	
Indole test	Positive
Methyl red test	Positive
Voges-Proskauer test	Positive
Citrate utilisation test	Negative
Catalase Test	Positive
Oxidase test	Positive
Starch hydrolysis	Positive

**Table 2:** Zone of inhibition of *W.* *coagulans* strain LMG S-31876 against various antibiotics.

Sl. No.	Antibiotic	Concentration (in µg)	Zone of Inhibition (in mm)
1.	Polymyxin-B	PB-300	30
2.	Amoxyclav	AMC-30	42
3.	Rifampicin	RIF-5	35
4.	Tetracycline	TE-30	44
5.	Oxacillin	OX-5	35
6.	Amikacin	AK-30	27
7.	Cefoxitin	CX-30	31
8.	Cefepime	CPM-30	33
9	Ceftazidime	CAZ-30	24
10.	Cefotaxime	CTX-30	25
11.	Chloramphenicol	C-30	34
12.	Cefdinir	CDR-5	37
13.	Penicillin g	P-10	18
14.	Moxifloxacin	MO-5	35
15.	Ampicillin	AMP-10	19
16.	Vancomycin	VA-30	22
17.	Ceftriaxone	CTR-30	30
18.	Neomycin	N-10	24
19.	Ofloxacin	OF-5	35
20.	Norfloxacin	NX-10	33
21.	Kanamycin	K-30	30
22.	Bacitracin	B-10	24
23.	Co-Trimoxazole	COT-25	13
24.	Methicillin	MET-10	37
25.	Streptomycin	S-10	22
26.	Levofloxacin	LE-5	35
27.	Erythromycin	E-15	11
28.	Clindamycin	CD-2	29
29.	Gentamycin	HLG-120	36
30.	Sterile disc	Control	0

**Table 3:** Antibacterial activity of *W. coagulans* strain LMG S-31876 against selected pathogenic bacterial strains.

Sl. No.	Test Pathogens	Zone of Inhibition (in mm)
1.	*Staphylococcus aureus* strain GCC_20MS	21
2.	*Mammaliicoccus sciuri* strain GCC_20RS	18
3	*Bacillus cereus* strain GCC_21R1	10
4.	*Bacillus nealsonii* strain GCC_21R8	14
5.	*Bacillus megaterium* strain GCC-SO1	14
6.	*Enterobacter bugandensis* strain GCC_21R10	18
7.	*Pseudomonas aeruginosa* strain GCC_19W1	20
8.	*Stenotrophomonas maltophilia* strain GCC_19W2	18
9.	*Achromobacter spanius* strain GCCSB1	20
10.	*Acinetobacter johnsonii* strain SB_SK	17

## Data Availability

The data that support the findings of this study are available in figshare.com (accessed on 25 July 2022) at https://doi.org/10.6084/m9.figshare.19487726.

## References

[1] Bergey D.H., Breed R.S., Murray E.G.D., Hitchens A.P. (1939). Manual of Determinative Bacteriology.

[2] Breed B.R., Murray E.G.D., Smith N.R. (1957). Bergey’s. Manual of Determinative Bacteriology.

[3] Gupta R.S., Patel S., Saini N., Chen S. (2020). Robust demarcation of 17 distinct Bacillus species clades, proposed as novel Bacillaceae genera, by phylogenomics and comparative genomic analyses: Description of Robertmurraya kyonggiensis sp. nov. and proposal for an emended genus Bacillus limiting it only to the members of the Subtilis and Cereus clades of species. Int. J. Syst. Evol. Microbiol..

[4] Shudong P., Guo C., Wu S., Cui H., Suo H., Duan Z. (2022). Bioactivity and metabolomics changes of plant-based drink fermented by *Bacillus coagulans* VHProbi C08. LWT.

[5] Lee B., Lee H., Jeong D.-W., Lee J.-H. (2015). A rapid isolation method for *Bacillus coagulans* from Rice Straw. Microbiol. Biotechnol. Lett..

[6] Konuray G., Erginkaya Z. (2018). Potential use of *Bacillus coagulans* in the food industry. Foods.

[7] Bang W.Y., Ban O.-H., Lee B.S., Oh S., Park C., Park M.-K., Jung S.K., Yang J., Jung Y.H. (2021). Genomic-, phenotypic-, and toxicity-based safety assessment and probiotic potency of *Bacillus coagulans* IDCC 1201 isolated from green malt. J. Ind. Microbiol. Biotechnol..

[8] Majeed M., Nagabhushanam K., Arumugam S., Majeed S., Ali F. (2018). *Bacillus coagulans* MTCC 5856 for the management of major depression with irritable bowel syndrome: A randomised, double-blind, placebo controlled, multi-centre, pilot clinical study. Food Nutr. Res..

[9] Majeed M., Nagabhushanam K., Natarajan S., Sivakumar A., Ali F., Pande A., Majeed S., Karri S.K. (2016). *Bacillus coagulans* MTCC 5856 supplementation in the management of diarrhea predominant Irritable Bowel Syndrome: A double blind randomized placebo controlled pilot clinical study. Nutr. J..

[10] Hun L. (2009). *Bacillus coagulans* significantly improved abdominal pain and bloating in patients with IBS. Postgrad. Med..

[11] Maity C., Gupta A.K., Saroj D.B., Biyani A., Bagkar P., Kulkarni J., Kulkarni J. (2021). Impact of a gastrointestinal stable probiotic supplement *Bacillus coagulans* LBSC on human gut microbiome modulation. J. Diet. Suppl..

[12] Jäger R., Shields K.A., Lowery R.P., De Souza E.O., Partl J.M., Hollmer C., Purpura M., Wilson J.M. (2016). Probiotic *Bacillus coagulans* GBI-30, 6086 reduces exercise-induced muscle damage and increases recovery. PeerJ.

[13] Anaya-Loyola M.A., Enciso-Moreno J.A., López-Ramos J.E., García-Marín G., Orozco Álvarez M.Y., Vega-García A.M., Mosqueda J., García-Gutiérrez D.G., Keller D., Pérez-Ramírez I.F. (2019). *Bacillus coagulans* GBI-30, 6068 decreases upper respiratory and gastrointestinal tract symptoms in healthy Mexican scholar-aged children by modulating immune-related proteins. Food Res. Int..

[14] Ratna Sudha M., Yelikar K.A., Deshpande S. (2012). Clinical study of *Bacillus coagulans* unique IS-2 (ATCC PTA-11748) in the treatment of patients with bacterial vaginosis. Indian J. Microbiol..

[15] Dolati M., Tafvizi F., Salehipour M., Movahed T.K., Jafari P. (2021). Inhibitory effects of probiotic *Bacillus coagulans* against MCF7 breast cancer cells. Iran. J. Microbiol..

[16] Baron M. (2009). A patented strain of *Bacillus coagulans* increased immune response to viral challenge. Postgrad. Med..

[17] Majeed M., Nagabhushanam K., Natarajan S., Arumugam S., Pande A., Majeed S., Ali F. (2016). A double-blind, placebo-controlled, parallel study evaluating the safety of *Bacillus coagulans* MTCC 5856 in healthy individuals. J. Clin. Toxicol..

[18] Tamang J.P., Shin D.-H., Jung S.-J., Chae S.-W. (2016). Functional properties of microorganisms in fermented foods. Front. Microbiol..

[19] Cao J., Yu Z., Liu W., Zhao J., Zhang H., Zhai Q., Chen W. (2020). Probiotic characteristics of *Bacillus coagulans* and associated implications for human health and diseases. J. Funct. Foods.

[20] Keller D., Verbruggen S., Cash H., Farmer S., Venema K. (2019). Spores of *Bacillus coagulans* GBI-30, 6086 show high germination, survival and enzyme activity in a dynamic, computer-controlled in vitro model of the gastrointestinal tract. Benef. Microbes.

[21] Schillinger U., Guigas C., Holzapfel W.H. (2005). In vitro adherence and other properties of lactobacilli used in probiotic yoghurt-like products. Int. Dairy J..

[22] Salvetti E., Orrù L., Capozzi V., Martina A., Lamontanara A., Keller D., Cash H., Felis G.E., Cattivelli L., Torriani S. (2016). Integrate genome-based assessment of safety for probiotic strains: *Bacillus coagulans* GBI-30, 6086 as a case study. Appl. Microbiol. Biotechnol..

[23] Nath S., Roy M., Sikidar J., Deb B., Sharma I., Guha A. (2021). Characterization and in-vitro screening of probiotic potential of novel Weissella confusa strain GCC_19R1 isolated from fermented sour rice. Curr. Res. Biotechnol..

[24] Khagwal N., Sharma P.K., Sharma D.C. (2014). Screening and evaluation of Lactobacillus spp. for the development of potential probiotics. Afr. J. Microbiol. Res..

[25] Bora P.S., Puri V., Bansal A.K. (2009). Physicochemical properties and excipient compatibility studies of probiotic *Bacillus coagulans* spores. Sci. Pharm..

[26] Cappuccino J.G., Sherman N. (1996). Microbiology: A Laboratory Manual.

[27] Green M.R., Sambrook J. (2012). Molecular Cloning: A Laboratory Manual.

[28] Nath S., Deb B., Sharma I. (2018). Isolation of toxic metal-tolerant bacteria from soil and examination of their bioaugmentation potentiality by pot studies in cadmium-and lead-contaminated soil. Int. Microbiol..

[29] Archer A.C., Halami P.M. (2015). Probiotic attributes of Lactobacillus fermentum isolated from human feces and dairy products. Appl. Microbiol. Biotechnol..

[30] Nath S., Sikidar J., Roy M., Deb B. (2020). In vitro screening of probiotic properties of Lactobacillus plantarum isolated from fermented milk product. Food Qual. Saf..

[31] Kõll P., Mändar R., Smidt I., Hütt P., Truusalu K., Mikelsaar R.-H., Shchepetova J., Krogh-Andersen K., Marcotte H., Hammarström L. (2010). Screening and evaluation of human intestinal lactobacilli for the development of novel gastrointestinal probiotics. Curr. Microbiol..

[32] Shivangi S., Devi P.B., Ragul K., Shetty P.H. (2020). Probiotic potential of Bacillus strains isolated from an acidic fermented food Idli. Probiotics Antimicrob. Proteins.

[33] Pan W.-H., Li P.-L., Liu Z. (2006). The correlation between surface hydrophobicity and adherence of Bifidobacterium strains from centenarians’ faeces. Anaerobe.

[34] Shangpliang H.N.J., Sharma S., Rai R., Tamang J.P. (2017). Some technological properties of lactic acid bacteria isolated from Dahi and Datshi, naturally fermented milk products of Bhutan. Front. Microbiol..

[35] Xu H., Jeong H.S., Lee H.Y., Ahn J. (2009). Assessment of cell surface properties and adhesion potential of selected probiotic strains. Lett. Appl. Microbiol..

[36] Rastogi S., Mittal V., Singh A. (2019). In vitro evaluation of probiotic potential and safety assessment of Lactobacillus mucosae strains isolated from Donkey’s lactation. Probiotics Antimicrob. Proteins.

[37] Baccer R., Kirby W.M., Kirby W.M., Turck M. (1966). Antibiotic susceptibility testing by standard single disc diffusion method. Am. J. Clin. Pathol..

[38] CLSI (2021). Performance Standards for Antimicrobial Susceptibility Testing.

[39] Hussein E.I., Jacob J.H., Shakhatreh M.A.K., Al-Razaq M.A.A., Juhmani A.-S.F., Cornelison C.T. (2018). Detection of antibiotic-producing Actinobacteria in the sediment and water of Ma’in thermal springs (Jordan). Germs.

[40] Nath S., Sinha A., Singha Y.S., Dey A., Bhattacharjee N., Deb B. (2020). Prevalence of antibiotic-resistant, toxic metal-tolerant and biofilm-forming bacteria in hospital surroundings. Environ. Anal. Health Toxicol..

[41] Raveschot C., Cudennec B., Deracinois B., Frémont M., Vaeremans M., Dugersuren J., Demberel S., Drider D., Dhulster P., Coutte F. (2020). Proteolytic activity of Lactobacillus strains isolated from Mongolian traditional dairy products: A multiparametric analysis. Food Chem..

[42] Aspri M., Bozoudi D., Tsaltas D., Hill. C., Papademas. P. (2017). Raw donkey milk as a source of Enterococcus diversity: Assessment of their technological properties and safety characteristics. Food Control..

[43] do Espirito-Santo A.P., Mouquet-Rivier C., Humblot C., Cazevieille C., Icard-Vernière C., Soccol C.R., Guyot J.-P. (2014). Influence of cofermentation by amylolytic Lactobacillus strains and probiotic bacteria on the fermentation process, viscosity and microstructure of gruels made of rice, soy milk and passion fruit fiber. Food Res. Int..

[44] Abdhul K., Ganesh M., Shanmughapriya S., Vanithamani S., Kanagavel M., Anbarasu K., Natarajaseenivasan K. (2015). Bacteriocinogenic potential of a probiotic strain *Bacillus coagulans* [BDU3] from Ngari. Int. J. Biol. Macromol..

[45] Altun G.K., Erginkaya Z. (2021). Identification and characterization of *Bacillus coagulans* strains for probiotic activity and safety. LWT.

[46] Singhal N., Singh N.S., Mohanty S., Singh P., Virdi J.S. (2019). Evaluation of Probiotic Characteristics of Lactic Acid Bacteria Isolated from Two Commercial Preparations Available in Indian Market. Indian J. Microbiol..

[47] Sharma S., Kandasamy S., Kavitake D., Shetty P.H. (2018). Probiotic characterization and antioxidant properties of Weissella confusa KR780676, isolated from an Indian fermented food. LWT.

[48] Mortuza T. (2016). Isolation and Identification of Microbes from Various Fruit Juices Made and Sold for Immediate Consumption at Home and in the Market of Dhaka City.

[49] Shinde T., Vemuri R., Shastri M.D., Perera A.P., Tristram S., Stanley R., Eri R. (2019). Probiotic *Bacillus coagulans* MTCC 5856 spores exhibit excellent in-vitro functional efficacy in simulated gastric survival, mucosal adhesion and immunomodulation. J. Funct. Foods.

[50] Ruiz L., Margolles A., Sánchez B. (2013). Bile resistance mechanisms in Lactobacillus and Bifidobacterium. Front. Microbiol..

[51] Bernet-Camard M.F., Coconnier M.H., Hudault S., Servin A.L. (1996). Differentiation-associated antimicrobial functions in human colon adenocarcinoma cell lines. Exp. Cell Res..

[52] Ragul K., Syiem I., Sundar K., Shetty P.H. (2017). Characterization of probiotic potential of Bacillus species isolated from a traditional brine pickle. J. Food Sci. Technol..

[53] de Souza B.M.S., Borgonovi T.F., Casarotti S., Todorov S. (2019). Lactobacillus casei and Lactobacillus fermentum strains isolated from mozzarella cheese: Probiotic potential, safety, acidifying kinetic parameters and viability under gastrointestinal tract conditions. Probiotics Antimicrob. Proteins.

[54] Shakirova L., Grube M., Gavare M., Auzina L., Zikmanis P. (2013). Lactobacillus acidophilus La5 and Bifidobacterium lactis Bb12 cell surface hydrophobicity and survival of the cells under adverse environmental conditions. J. Ind. Microbiol. Biotechnol..

[55] Krausova G., Hyrslova I., Hynstova I. (2019). In vitro evaluation of adhesion capacity, hydrophobicity, and auto-aggregation of newly isolated potential probiotic strains. Fermentation.

[56] Soni R., Keharia H., Dunlap C., Pandit N., Doshi J. (2022). Functional annotation unravels probiotic properties of a poultry isolate, Bacillus velezensis CGS1. 1. LWT.

[57] Anandharaj M., Sivasankari B. (2014). Isolation of potential probiotic Lactobacillus oris HMI68 from mother’s milk with cholesterol-reducing property. J. Biosci..

[58] Saroj D.B., Gupta A.K. (2020). Genome based safety assessment for *Bacillus coagulans* strain LBSC (DSM 17654) for probiotic application. Int. J. Food Microbiol..

[59] Sui L., Zhu X., Wu D., Ma T., Tuo Y., Jiang S., Qian F., Mu G. (2020). In vitro assessment of probiotic and functional properties of *Bacillus coagulans* T242. Food Biosci..

[60] Kim Y.-S., Lee J., Heo S., Lee J.-H., Jeong D.-W. (2021). Technology and safety evaluation of *Bacillus coagulans* exhibiting antimicrobial activity for starter development. LWT.

[61] Choudhury P., Bhunia B. (2015). Industrial application of lipase: A review. Biopharm. J..

[62] Ray A. (2012). Application of lipase in industry. Asian J. Pharm. Technol..

[63] Wang Y., Cao W., Luo J., Qi B., Wan Y. (2019). One step open fermentation for lactic acid production from inedible starchy biomass by thermophilic *Bacillus coagulans* IPE22. Bioresour. Technol..

[64] Bailon-Salas A.M., Ordaz-Díaz L.A., Valle-Cervantes S., López-Miranda J., Urtiz-Estrada U., Páez-Lerma J.B., Rojas-Contreras J.A. (2018). Characterization of Culturable Bacteria from Pulp and Paper Industry Wastewater, with the Potential for Degradation of Cellulose, Starch, and Lipids. BioResources.

